# Patient and Public Involvement (PPI) in outcome selection in breast cancer and nephrology trials

**DOI:** 10.1186/s13063-022-06980-9

**Published:** 2023-02-07

**Authors:** Ciara Buckley, Shaun Treweek, Lynn Laidlaw, Frances Shiely

**Affiliations:** 1 Trials Research and Methodologies Unit, HRB Clinical Research Facility, University College Cork, Cork, Ireland; 2 School of Public Health, University College Cork, Cork, Ireland; 3 HRB Trials Methodology Research Network (TMRN), University College Cork, Cork, Ireland; 4 Health Services Research Unit, University of Aberdeen, Aberdeen, UK; 5 Patient and Public Contributor, Health Service Research Unit, Aberdeen, UK

**Keywords:** Trial methodology, Outcomes, Clinical trials, Patient and public involvement/PPI

## Abstract

We recently reported that according to patients and healthcare professionals in breast cancer and nephrology trials, teams conducting the trials got their choice of primary outcome wrong (72% of the time) more often than they got it right (28% of the time). A Patient and Public Involvement (PPI) representative, co-author of this letter, asked (on Twitter) whether PPI contributors had been involved in the design of the original trials and by extension the outcome selection. The purpose of this study was to answer this question.

## Introduction

In a recently published study, we investigated how important patients (*n* = 30) and healthcare professionals (*n* = 12), with experience in the clinical areas of breast cancer and nephrology, consider the outcomes (particularly the primary outcome) measured in a random selection of 20 breast cancer and 24 nephrology published randomised controlled trials [1]. Primary outcomes are deemed the most important outcomes by trial investigators, and the primary outcome is used to determine the sample size for the trial [2] as well as being the main way to judge whether the intervention is effective or not. Secondary outcomes aid with decision-making by providing additional information but are considered less important [1].

We found that according to the patients and healthcare professionals, teams conducting breast cancer and nephrology trials got their choice of primary outcome wrong (72% of the time) more often than they got it right (28% of the time) [1]. A Patient and Public Involvement (PPI) representative, co-author of this letter (LL), asked (on Twitter) whether PPI contributors had been involved in the design of the original trials and by extension the outcome selection. We were unable to answer this question.

The purpose of this short research piece is to answer that question. In addition, we wanted to see if the outcome selection in trials involving PPI contributors was more agreeable to patients and healthcare professionals compared to trials that did not have PPI contributors.

## Methods

The methods used to randomly select the original 44 trials have been described previously [1]. For the current study, the protocol for each of the 44 trials was sought. Trial registration numbers were found for each trial by reviewing the original published research paper and searching clinical trial registries. Any supplementary documents, for example, audio files, diagrams, disclosure forms and tables, available online for trials were also examined for any information related to PPI in the trial.

Trial protocols were readily available for 25 of the research papers: 13 for the breast cancer trials and 12 for the nephrology trials. For the remaining 19 trials, email contact for the corresponding author was located in the published research paper, and contact was made with each corresponding author. Of the 19 corresponding authors emailed, six replied (four replied providing the trial protocol while two did not have access to the protocol and could only provide some information regarding their study that they believed would be useful to our research objectives), nine did not respond, one had moved institute and was not in a position to supply the protocol and the final three could not be contacted as their email addresses were no longer valid. Extracted data were recorded in a MS Excel spreadsheet.

## Results

In total, the 44 original publications and 29 associated protocols were examined for this study: 17 for the breast cancer trials and 12 for the nephrology trials. There were also 48 supplementary files examined: 25 for the breast cancer trials and 23 for the nephrology trials. PPI information on the breast cancer trials and nephrology trials are presented in Tables 1 and 2, respectively.

**Table 1:** PPI information associated with the 20 breast cancer trials

Trial IDs	Trial registration number	PPI information in study publication	PPI Information in the study protocol	PPI information in supplementary files
Trial 1	NCT01958021	No	No	No
Trial 2	NCT00433589	“The dichotomous cutoff was chosen by a consensus of all TRANSBIG partners, including patient representatives, to define a situation in which the absolute benefit of chemotherapy would balance its associated side effects”.	Steering Committee: representatives from EORTC, TRANSBIG, IDDI, NKI, Agendia, IGR, FNCLCC, NCC/Gs and patient’s representative organizations.	No
Trial 3	NCT00878709	No	No	No
Trial 4	NCT00193778	No	No	No
Trial 5	NCT00402519	No	No	No
Trial 6	NCT00053898	No	No	No
Trial 7	NCT00408408	No	No	No
Trial 8	NCT00433420	No	No	No
Trial 9	NCT00310180	No	No	No
Trial 10	NCT01602380	No	No	No
Trial 11	ISRCTN37546358	No	No	No
Trial 12	NCT00600340	No	No	No
Trial 13	NCT01419197	No	The corresponding author has moved institute and was not in a position to supply the protocol.	No
Trial 14	NCT00039546	No	No	No
Trial 15	NCT01093235	No	No	No
Trial 16	NCT01610284	No	No	No
Trial 17	ISRCTN91879928	No	A Public and Patient Involvement event was hosted at Queen Mary University of London in January 2020, to collaboratively discuss with breast cancer research participants their views on the study procedures specifically relating to the collection of identifiable data for use in future analyses. An open discussion on how participants felt about the study procedures specifically on long-term follow-up data collection was initiated. There was a very positive response where patients felt happy to be included in such long-term follow-up research, their data to be collected as described in this study protocol and their data used for analyses. Patients commented on the appropriateness to conduct analyses with the use of long-term follow-up data using their personal identifiable data for linkage with these registries. We also made sure to highlight that under no circumstances identifiable data would be released to a third party and that all data will always remain in a secure and locked environment.	No
Trial 18	NCT01772472	No	No	No
Trial 19	NCT01740427	No	No	No
Trial 20	NCT00002851	No	No	No

**Table 2:** PPI information associated with the 24 nephrology trials selected

Trial IDs	Trial registration number	PPI information in study publication	PPI information in the study protocol	PPI information in supplementary files
Trial 1	NCT01351675	No	No	No
Trial 2	NCT00598273	No	No	No
Trial 3	NCT00081731	No	No	No
Trial 4	NCT03071263	No	No	No
Trial 5	ISRCTN45967602	No	No	No
Trial 6	NCT02476253	No	No	No
Trial 7	NCT01862419	No	No	No
Trial 8	ISRCTN99959692	No	No	No
Trial 9	JPRN-C000000008	No	No	No
Trial 10	NCT02345057	No	No	No
Trial 11	NCT02332824	No	No	No
Trial 12	NCT00396032	No	No	No
Trial 13	NCT00402168	No	No	No
Trial 14	NCT01208818	No	No	No
Trial 15	NCT00463294	No	No	No
Trial 16	NCT01767883	No	No	No
Trial 17	NCT01683409	No	No	No
Trial 18	CTI-111433	No	No	No
Trial 19	NCT01320202	No	No	No
Trial 20	ISRCTN11958993	No (steering committee members do not appear to be patients or patient representatives)	No	No
Trial 21	NCT01691053	No	No	No
Trial 22	NCT00317239	No	No	No
Trial 23	NCT01493024	Yes (interviews with some patients to test enrolment efficiency but patients were not involved in outcome selection)^a^	No. “In generating the protocol, we did not specifically ask for patient input, since an existing drug (sodium polystyrene sulfonate) is ingested by patients on a similar schedule and for the same reasons. That drug is not very effective and has known side effects. Our goal was to make the drug much more effective and with fewer side effects. I felt that all of my patients taking the older drug would agree with this goal and the protocol”.	No
Trial 24	NCT02141672	No	No	No

^a^No mention of PPI in study publication—information obtained from the author when requesting the protocol

We found no evidence in any of the main trial publications, protocols or any other available trial documents that there was PPI in selecting the outcomes for any of the 20 breast cancer trials or 24 nephrology trials.

### PPI information in the original main trial publication

Of the 20 breast cancer publications, two made reference to PPI, one in the results and the other in the discussion. The first mentioned patient involvement in the decision to “…define a situation in which the absolute benefit of chemotherapy would balance its associated side effects” and the second mentioned “decisions made by either patients or physicians” to discontinue treatment due to adverse events. Neither is related to outcome selection.

Of the 24 nephrology publications, there was no mention of PPI. However, the corresponding author of one of the papers stated that there were in fact some PPI as there were “interviews with some patients to test enrolment efficiency but patients were not involved in outcome selection”.

### PPI information in the study protocol

Only two of the available 17 breast cancer protocols mentioned PPI. The protocol for one study explicitly mentioned that there was PPI membership on the Trial Steering Committee. The protocol for the second study detailed a PPI event hosted specifically to discuss research participants’ views on the study procedures relating to the collection of identifiable data and follow-up data.

Of the 12 nephrology protocols, there was no mention of PPI. The corresponding author of one protocol wrote in their email, “In generating the protocol we did not specifically ask for patient input, since an existing drug (sodium polystyrene sulfonate) is ingested by patients on a similar schedule and for the same reasons. That drug is not very effective and has known side effects. Our goal was to make the drug much more effective and with fewer side effects. I felt that all of my patients taking the older drug would agree with this goal and the protocol”.

### PPI information in supplementary files

There was no PPI information given in any of the other available study files.

## Discussion

PPI in research has the potential to benefit clinical trials by ensuring that the trial design is relevant, ethical and the trial is attractive to possible future participants [3]. PPI makes it more likely that the trial results are relevant to those impacted by a condition and it can also improve recruitment and retention [4].

Our findings speak volumes about the reality of “patient/person-centred care” and shared decision-making in clinical trials—researchers make the important decisions and do not see the need to check in with the patient. The comment from one corresponding author, “I felt that all of my patients taking the older drug would agree with this goal and the protocol”, exemplifies this. The researcher knows best. Our results confirm the mismatch between policy and the delivery of healthcare along with the way we produce evidence [5].

We need to ask the question, “Why are the opinions of patients overlooked when the primary purpose of a clinical trial is to offer patients a treatment/therapy that may improve their health and ultimately quality of life?” Excluding those with the greatest stake in the success of a treatment or therapy is a mistake and we argue it produces “bad” research. We recently published a paper on the continuing scandal of poor medical research [6]. We strongly feel that the lack of PPI representation in trial methodology and conduct is adding to this scandal. Patients must be given the opportunity to become involved in all aspects of the trial process to ensure research is relevant, can achieve its full potential for patients and the public and reduce the chance that scarce resources are wasted.

In order to enable authentic involvement, a “space to talk” and a “space to change” must be provided. These spaces welcome the opportunity for all to share dialogue, deal with any tension or disagreements that may arise between PPI contributors and researchers and also adapt in response to contributor feedback in a way that respects and values all types of expertise equally [7].

Our findings raise questions about the way that PPI is conducted, especially in relation to power and agency. Who decides how involved any PPI group/contributor will be in a trial? [8, 9]. We encourage the trial teams to refer to GRIPP2 [10], international guidance on reporting of patient and public involvement in health and social care research, when planning their trial. GRIPP2 is a reporting guideline, typically used when the trial is completed. However, using GRIPP2 at the planning and design stage will show the trial team that PPI in all aspects of the trial is encouraged, even expected.

We have highlighted the lack of transparency in relation to how PPI is reported. The lack of PPI information is palpable in the 44 trials included in this study. There are no PPI co-authors, hardly surprising given there was very little PPI involvement generally and none in selecting the outcomes for the trials. We recommend that in future trials, if PPI representatives are part of the trial team, their input should be acknowledged in a meaningful way and co-authorship should be standard practice. This has been previously discussed in the literature [10, 11].

Our original study found that patients and healthcare professionals disagreed with the trial team’s choice of primary outcome 72% of the time [1]. It does not matter how robust the methodology of a trial is or how experienced the research team is, if the outcome that is most important to people living with a disease (and those treating it) is not measured, or not measured to a sufficient degree of certainty, then it is quite possible that the results will be considered irrelevant. We need to ensure that what we measure is meaningful as well as measurable for the trial to support improved healthcare decision-making.

Our 44 included trials are relatively recent (the earliest was published in 2010), but the body of literature supporting the inclusion of patients and the public precedes these [12–15]. According to an editorial as far back as 2008 [16], PPI had gained increasing recognition for its potential in various aspects of healthcare activity both in the UK and internationally over the previous decade. The UK standards for public involvement in research were published in 2019, and in our study of 44 international trials, all fall short of these standards [17].

## Data Availability

The 44 protocols used are hyperlinked in the tables.
